# Aerosol characteristics and impacts on weather and climate over the Tibetan Plateau

**DOI:** 10.1093/nsr/nwz184

**Published:** 2019-11-14

**Authors:** Chuanfeng Zhao, Yikun Yang, Hao Fan, Jianping Huang, Yunfei Fu, Xiaoye Zhang, Shichang Kang, Zhiyuan Cong, Husi Letu, Massimo Menenti

**Affiliations:** 1 State Key Laboratory of Earth Surface Processes and Resource Ecology, and College of Global Change and Earth System Science, Beijing Normal University, China; 2 Collaborative Innovation Center for Western Ecological Safety, College of Atmospheric Sciences, Lanzhou University, China; 3 School of Earth and Space Sciences, University of Science and Technology of China, China; 4 Institute of Atmospheric Composition, Chinese Academy of Meteorological Sciences, China; 5 State Key Laboratory of Cryospheric Sciences, Northwest Institute of Eco-Environment and Resources, Chinese Academy of Sciences, China; 6 Institute of Remote Sensing and Digital Earth, Chinese Academy of Sciences, China

With the most massive snow and ice globally between 60°S and 60°N, the Tibetan Plateau (TP), also called the third pole of the world, plays significant roles in the weather, climate and lives in South Asia and East Asia and even the world [1–3]. Investigating the variation of weather and the climate system over the TP has long been carried out, which is significant for fresh water supplies for over 1.4 billion people in Asia. Actually, the ‘Third Pole Environment’ program was initiated to call international efforts to understand both climate and environment changes on the plateau [1], which has already shown rapid warming and intensified cryospheric melt and water cycle.

Previous studies have indicated that the clouds and radiation are particularly sensitive to aerosols over pristine regions [4], such as the Arctic and TP. Thus, aerosols could play an essential role in the weather and climate system over the TP by directly absorbing and scattering solar radiation and indirectly modifying the cloud properties, particularly when absorbing aerosols such as black carbon (BC) fall into snow at the surface. Actually, it has been reported that the atmospheric aerosols are highly related to the shrinkage of glaciers and snow over the TP [3].

Increasing attention has been paid to the TP for aerosol characteristics and impacts [3,5]. Particularly, the TP is surrounded by East Asia and South Asia—two regions with almost the world's most serious anthropogenic emissions and a world-known Taklamakan Desert. Studies have shown that the anthropogenic emissions in South Asia and dust from the Taklamakan Desert can be transported to the TP region [3,6]. Moreover, large amounts of light-absorbing aerosols (LAAs) such as BC have been found over the northern Indo-Gangetic plain, implying non-negligible transport of LAAs from India to the TP [7].

## AEROSOL CHARACTERISTICS

**Figure 1.**
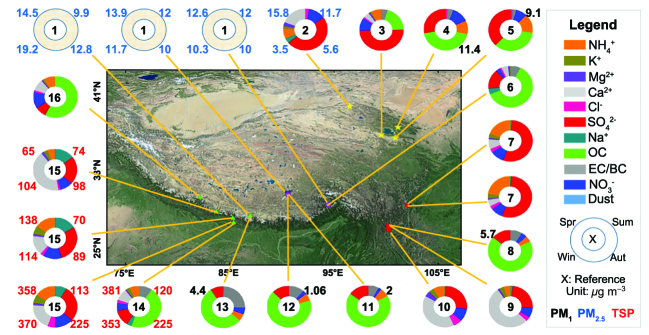
A review of aerosol characteristics at various ground sites or regions over the Tibetan Plateau based on 16 previous studies. Spr, Sum, Aut and Win represent the four seasons of spring, summer, autumn and winter, respectively. The black, sky-blue and red numbers around the circles denote PM_1_, PM_2.5_ and TSP with unit μg m^−3^, respectively. OC and EC/BC represent organic carbon and elemental carbon/black carbon, respectively. X indicates the serial number for the previous study reference, which is also listed in Supplementary Table 1.

Figure 1 summarizes the aerosol characteristics measured from surface sites over the TP reported by previous studies for different time periods. The site names, locations, observation periods, along with the literature have been listed in Supplementary Table 1. There are 14 surface sites, with most in the south, southeast and northeast TP. No surface sites exist in the middle and northwest TP due to the high elevation and harsh climate, over which the aerosol characteristics are mainly derived from satellite observations. However, the satellite retrieval of aerosol properties is particularly difficult over the TP, due to the complicated anisotropic reflection of the land surface. The Multiangle Imaging SpectroRadiometer showed that the 7-year average aerosol optical depth (AOD) are 0.27, 0.25, 0.13 and 0.11 from spring to winter over the TP [5]. Although having low AOD compared to both East Asia and South Asia regions around, the aerosol particles over the TP are still influenced by the transport of aerosols from surrounding areas [6], particularly for marginal regions of the TP. For example, the daily averages of PM_2.5_ (particulate matter with aerodynamic diameters below 2.5 μm) were 18.2 ± 8.9, 14.5 ± 7.4, 11.9 ± 4.9 and 11.7 ± 4.7 μg m^−3^ at Qomolangma Station (QOMS, barren site), Nam Co (grassland site) and Southeastern Tibetan (SET, forest site), respectively, which are larger than the background values that are generally below 10 μg m^−3^ [8]. For some cases, the total aerosol mass concentration is even several times larger, since there is a large portion of dust aerosols over the TP, as indicated by the differences between PM_2.5_ and Total Suspended Particulate (TSP) in Fig. 1. Note that, in the central TP, the aerosol mass concentration is still at a fairly low level, as observed at the Nam Co station; thus, one should not expect too much pollution there.

Figure 1 demonstrates that the aerosol amount and type have apparent spatial and seasonal variations, implying influences from different sources. At the north TP, transported dust aerosols from Taklamakan Desert may accumulate and cause a high concentration of dust aerosols [6]. At the south TP, we can find a high fraction of organics, sulfate, dust (Ca^2+^) and BC, which is most likely caused by the transport of anthropogenic aerosols from India, likely through the mechanism of ‘up and over’ transport. A clear seasonal variation can be found for both the TSP and PM_2.5_ over the south TP, which is high in spring and/or winter, and low in summer. The relatively low TSP in summer should be associated with the strong precipitation scavenging. At the southeast TP, the aerosol types vary significantly among different studies, while it seems that organics and sulfate are dominant types. At the northeast TP, the dominant aerosol types are also sulfate and organics, with considerable contributions from dust. Figure 1 also shows that the PM_2.5_ is much higher in spring and summer over the northeast TP, which could be associated with the more strong dust events in those two seasons. In contrast, higher BC concentrations in snow were observed in the north TP than in the south TP [9]. However, this does not indicate that more BCs are transported into the north TP. Instead, this is more likely related to shallower snow depths and then dirtier snow in the north TP as a result of post-depositional processes (e.g. snowdrift or melting) [9]. Careful attention should be paid to the potential biases due to the uneven distribution of stations, which are mainly located in the marginal area of the TP.

Results above have shown that the major components of aerosols over the TP are dust, BC and sulfate/nitrate, while the dominant type varies with site. Anthropogenic BC and other man-made aerosols could also be increasing in recent years associated with the increased pollution in India. However, owing to the limited researches, sparse observation sites and short-term observations, it is still not very clear about the aerosol loading, source, composition, particle size and deposition, which need further study with more observations in future.

## AEROSOL IMPACTS ON WEATHER AND CLIMATE

**Figure 2.**
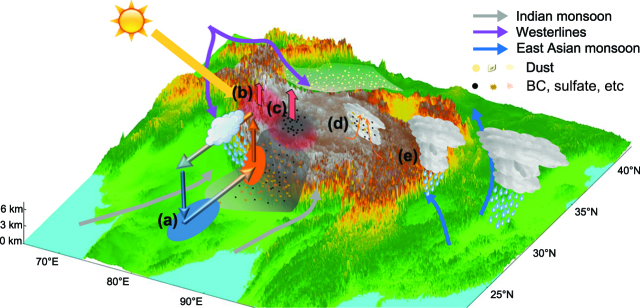
A schematic diagram that demonstrates the potential impacts of aerosols on weather and climate based on literature review: (a) the postponing and strengthening precipitation by aerosols from middle India to south of Himalaya mountains, proposed by [10]; (b) the ‘heat-pump’ effect proposed by [2]; (c) the enhanced ‘heat-pump’ effect by absorbing aerosols, proposed by multiple studies including [11]; (d) enhanced convective precipitation by aerosols that serve as cloud-condensation nuclei, which has been indicated by various studies including [14]; (e) enhanced convection could strengthen the precipitation events for downstream regions as shown by [12].

Figure 2 shows a schematic diagram that demonstrates how aerosols affect the weather and climate over the TP based on a literature review [10–13]. However, we should note that the confidence level about the aerosol impacts on weather and climate is even lower than the current knowledge about the aerosol characteristics over the TP.

The aerosols emitted over India could reduce solar radiation reaching the ground surface, making the surface warming less than previously [11]. Moreover, the LAAs would even further absorb solar radiation, making the near-surface air temperature more heated than that without. These two aerosol radiative effects would change the atmospheric thermal structure, causing the atmosphere to be more stable over most India regions and then reducing the frequencies of both clouds and precipitation. The aerosols and water vapors, which should have rained out over middle or south India regions, could be further transported to the southern slope of the TP along with enhanced latent heat energy, forming more clouds and stronger precipitation. This mechanism is demonstrated in Fig. 2a.

The polluted aerosols, including both BC and soluble particles, can even pass through the Himalayan mountains and reach internal regions of the TP, causing impacts on the formation and development of clouds and precipitation there. Previous studies [10] proposed the well-known ‘heat-pump’ effect of the TP, which is demonstrated in Fig. 2b.

LAAs reaching the TP, by absorbing more solar radiation at/near the surface, could enhance the ‘heat-pump’ effect, likely making stronger convection over the TP and more atmospheric constituents transported to/through the TP, which is demonstrated in Fig. 2c. This mechanism has been proposed by multiple studies, such as that by Lau and Kim [12]. Those studies suggested that the increased net surface solar radiation absorption by LAAs would cause rapid snowmelt over the TP and warming of the upper troposphere, followed by enhanced low-level south-westerlies and increased dust loading over the Himalayas-Indo-Gangetic Plain. This effect could further cause an enhancement of the Tibetan anticyclone and the development of an anomalous Rossby wave train over East Asia, leading to northward displacement and intensification of the Mei-Yu rain belt [12].

Not only the LAAs at the surface could enhance the ‘heat-pump’ effect; the LAAs at the surface along with other types of aerosols in the atmosphere could invigorate the convective clouds over the TP (e.g. [14]). Cloud-resolving model analysis exhibited that increasing the aerosol concentration generally enhances the cloud core updraft and maximum updraft [14], intensifying the convection and precipitation over the TP, which is demonstrated in Fig. 2d. The enhanced convection not only brings more precipitation over the TP, but also likely affects the rainfall in downstream regions. It is found that convective clouds can be transported downstream to regions including south China, causing strong convective precipitation, as demonstrated in Fig. 2e [13]. Similarly, the enhancement of convection by LAAs over the TP could also result in heavier precipitation over downstream regions [13].

By combining the previous studies, we proposed a potential teleconnection between Indian pollution and convective precipitation over the TP and South China. Anthropogenic aerosols from India, along with the dust aerosols from surrounding regions, would enhance the convective precipitation over the TP and then increase the precipitation over downstream areas in China. A time lag might exist between India pollution and precipitation over the TP and South China. However, considering that multiple factors could affect the clouds and precipitation, this teleconnection could be difficult to identify from observations clearly. Moreover, the mechanisms proposed above could be hardly evaluated from precipitation observations due to the multiple competing influential factors, such as global warming, glaciers, aerosols and so on. For example, we know that the aerosol effects coexist and somehow compete with the global-warming effect [2]. The global-warming climate background and/or multiple-decade climate-system oscillations have made surface pressure over the TP increase significantly and heating of the surface and atmosphere weakened [15], which may affect the summer precipitation downstream. By enhancing the convection and increasing the surface temperature, LAAs may alleviate/counter the effects caused by the global-warming background.

## REMAINING CHALLENGES

Significant progress in aerosol characteristics and aerosol weather and climate impacts have been obtained over the TP during the recent 20 years. Particularly, the anthropogenic aerosol pollution in India could result in more aerosols, water and energy transported to the TP, a more significant ‘heat-pump’ effect, and stronger convection and precipitation in the TP and downstream regions. However, grand challenges still remain, which include but are not limited to the following six issues.

More dense and reliable long-period observations are highly demanded, particularly over the core region of the TP, including more accurate satellite retrievals, long-term collection of more ground-site observations and vertical profile information about the aerosol and cloud characteristics, which could provide us with a more comprehensive understanding about the weather and climate over and around the TP.

Because of the heterogeneity and temporal variability of land-surface conditions over the TP, accurate retrieval of AOD, aerosol type and properties requires better information on the vertical distribution of aerosols, in addition to accurate knowledge of surface spectral bidirectional reflectance.

Dust may undergo long-distance transport from the upper level over the TP to downstream. It is necessary to understand how this long-distance transport may affect the weather and climate downstream. It is also necessary to pay more attention to how aerosols (especially dust) interact with clouds under the environment of low-aerosol concentration.

The buildup of greenhouse gases, the change of sea-surface temperature and the change in surface conditions, including glacier and snow cover, also affect the weather and climate, making it complicated and challenging to identify the impacts of aerosols over the TP region, which may need further observations and improved modeling.

Cross-cutting studies are strongly demanded. Putting cloud dynamics, climate dynamics and environmental science together can greatly improve our understanding of the impact of aerosols on weather and climate over the TP.

The teleconnection proposed here, along with other potential teleconnections such as among the three poles, needs further observational evidence and more in-depth physical and model understanding.

## Supplementary Material

nwz184_Supplemental_File

## References

[1] Yao T , Thompson L, Mosbrugger V et al. Environ Dev 2012; 3: 52–64.

[2] Wu G , Li Z, Fu C et al. Sci China Earth Sci 2016; 59: 1–16.

[3] Kang S , Zhang Q, Qian Y et al. Natl Sci Rev 2019; 6: 796–809. 10.1093/nsr/nwz031 PMC8291388 34691935

[4] Garrett T , Zhao C. Nature 2006; 440: 787–9. 16598255 10.1038/nature04636

[5] Xia X , Wang P, Wang Y et al. Geophys Res Lett 2008; 35: L16804.

[6] Huang J , Minnis P, Yi Y et al. Geophys Res Lett 2007; 34: 529–38.

[7] Ramanathan V , Chung C, Kim D et al. Proc Natl Acad Sci USA 2005; 102: 5326–33. 15749818 10.1073/pnas.0500656102 PMC552786

[8] Liu B , Cong Z, Wang Y et al. Atmos Chem Phys 2017; 17: 449–63.

[9] Zhang Y , Kang S, Sprenger M et al. The Cryosphere 2018; 12: 413–31.

[10] Wu G , Liu Y, Wang T et al. J Hydrometeor 2007; 8: 770–89.

[11] Lau K , Ramanathan V, Wu G et al. Bull Amer Meteor Soc 2008; 89: 369–83.

[12] Lau K , Kim K. Atmosphere 2018; 9: 438. 32454985 10.3390/atmos9110438 PMC7243248

[13] Zhao Y , Xu X, Ruan Z et al. Meteorol Atmos Phys 2019; 131: 697–712.

[14] Zhou X , Bei N, Liu H et al. Atmos Chem Phys 2017; 17: 7423–34.

[15] Yang K , Wu H, Qin J et al. Glob Planet Change 2014; 112: 79–91.

