# Supplementary material for: Aerosol characteristics and impacts on weather and climate over the Tibetan Plateau
Source: Natl Sci Rev. 2019 Nov 14;7(3):492–5. doi: 10.1093/nsr/nwz184 (PMC8288843; doi:10.1093/nsr/nwz184)
Supplement: nwz184_Supplemental_File [file nwz184_supplemental_file.docx]

Supplementary information for

**Aerosol characteristics and impacts on weather and climate over the Tibetan Plateau**

Chuanfeng Zhao^1^, Yikun Yang^1^, Hao Fan^1^, Jianping Huang^2^, Yunfei Fu^3^, Xiaoye Zhang^4^, Shichang Kang^5^, Zhiyuan Cong^5^, Husi Letu^6^, Massimo Menenti^6^

Table S1. The observation period, site name and location for those shown in Figure 1. SET, QOMS, and QSS denote Southeastern Tibetan, Qomolangma Station, and Qilian Shan Station, respectively.

| No. | Reference | Observation period | Site Name | Location |
| --- | --- | --- | --- | --- |
| 1 | Liu et al., 2017 | 2011-2013 | SET (Lulang) | 29.46°N, 94.44°E |
|  |  |  | Nam Co | 30.77°N, 94.95°E |
|  |  |  | QOMS | 28.36°N, 86.95°E |
| 2 | Xu et al., 2014 | 2010/7/16-2011/7/28 | QSS | 39.50°N, 96.51°E |
| 3 | Li et al., 2013 | 2010/7/3-2010/8/26 | Bird Island | 36.98°N, 99.90°E |
| 4 | Du et al., 2015 | 2013/9/5-2013/10/15 | Menyuan | 37.61°N, 101.26°E |
| 5 | Zhang et al., 2019 | 2017/7/1-2017/7/31 | Waliguan | 36.28°N, 100.90°E |
| 6 | Zhao et al., 2013 | 2008/7/16-2009/7/26 | Lulang (SET) | 29.46°N, 94.44°E |
| 7 | Zhao et al., 2009 | 2005/12-2006/11 | GongGa | 29.65°N, 102.12°E |
| 8 | Zheng et al., 2017 | 2015/3/22-2015/4/14 | Mt.Yulong | 27.20°N, 100.20°E |
| 9 | Zhang et al., 2012 | 2010/1/24-2010/2/27 | Mt.Yulong | 27.10°N, 100.19°E |
| 10 | Zhang et al., 2011 | 2008/12-2009/1 | Lijiang | NA |
| 11 | Xu et al., 2018 | 2015/6/1-2015/6/30 | Nam Co | 30.77°N, 94.95°E |
| 12 | Wang et al., 2017 | 2015/5/30-2015/6/30 | Nam Co | 30.77°N, 94.98°E |
| 13 | Zhang et al., 2018 | 2016/4/12-2016/5/12 | QOMS | 28.36°N, 86.95°E |
| 14 | Wan et al., 2019 | 2013/4-2014/4 | Bode | 27.67°N, 85.38°E |
| 15 | Tripathee et al., 2017 | 2013/4/4-2014/3/31 | Dhunche | 28.11°N, 85.30°E |
|  |  |  | Jomsom | 28.76°N, 83.71°E |
|  |  |  | Bode | 27.67°N, 85.38°E |
| 16 | Xu et al., 2013 | Summer in 2019 - Spring in 2010 | Jima Yangzong Glacier | 30.21°N, 82.14°E |

References listed in Table S1:

1. Liu B, Cong Z and Wang Y et al. Background aerosol over the Himalayas and Tibetan Plateau: observed characteristics of aerosol mass loading. *Atmos Chem Phys* 2017; **17**: 449-63.
2. Xu J, Wang Z and Yu G et al. Characteristics of water soluble ionic species in fine particles from a high altitude site on the northern boundary of Tibetan Plateau: Mixture of mineral dust and anthropogenic aerosol. *Atmos Res* 2014; **143**: 43-56.
3. Li J, Wang G and Wang X et al. Abundance, composition and source of atmospheric PM_2.5_ at a remote site in the0020Tibetan Plateau, China. *Tellus B* 2013, 10.3402/tellusb.v65i0.20281.
4. Du W, Sun Y and Xu Y et al. Chemical characterization of submicron aerosol and particle growth events at a national background site (3295 m a.s.l.) on the Tibetan Plateau. *Atmos Chem Phys* 2015; **15**: 10811-24.
5. Zhang X, Xu J and Kang S et al. Chemical characterization and sources of submicron aerosols in the northeastern Qinghai–Tibet Plateau: insights from high-resolution mass spectrometry. *Atmos Chem Phys* 2019; **19**: 7897-911.
6. Zhao Z, Cao J and Shen Z et al. Aerosol particles at a high-altitude site on the Southeast Tibetan Plateau, China: Implications for pollution transport from South Asia. *J Geophys Res-Atmos* 2013; **118**: 11360-75.
7. Zhao Y, Wang Y and Wen T et al. Observation and Analysis on Water-soluble Inorganic Chemical Compositions of Atmospheric Aerosol in Gongga Mountain (in Chinese). *Environmental Science* 2009; **30**: 9-13.
8. Zheng J, Hu M and Du Z et al. Influence of biomass burning from South Asia at a high-altitude mountain receptor site in China. *Atmos Chem Phys* 2017; **17**: 6853-64.
9. Zhang N, Cao J and Ho K et al. Chemical characterization of aerosol collected at Mt. Yulong in wintertime on the southeastern Tibetan Plateau. *Atmos Res* 2012; **107**: 76-85.
10. Zhang N, He Y and Wang C et al. Chemical Characteristic of Water-Soluble Ions in Total Suspended Particles (TSP) at Lijiang Winter Time (in Chinese). *Environmental Science* 2011; **32**: 619-25.
11. Xu J, Zhang Q and Shi J et al. Chemical characteristics of submicron particles at the central Tibetan Plateau: insights from aerosol mass spectrometry. *Atmos Chem Phys* 2018; **18**: 427-43.
12. Wang J, Zhang Q and Chen M et al. First Chemical Characterization of Refractory Black Carbon Aerosols and Associated Coatings over the Tibetan Plateau (4730 m a.s.l). *Environ Sci Technol* 2017; **51**: 14072-82.
13. Zhang X, Xu J and Kang S et al. Chemical characterization of long-range transport biomass burning emissions to the Himalayas: insights from high-resolution aerosol mass spectrometry. *Atmos Chem Phys* 2018; **18**: 4617-38.
14. Wan X, Kang S and Rupakheti M et al. Molecular characterization of organic aerosols in the Kathmandu Valley, Nepal: insights into primary and secondary sources. *Atmos Chem Phys* 2019; **19**: 2725-47.
15. Tripathee L, Kang S and Rupakheti D et al. Chemical characteristics of soluble aerosols over the central Himalayas: insights into spatiotemporal variations and sources. *Environ. Sci Pollut Res* 2017; **24**: 24454-72.
16. Xu J, Zhang Q and Li X et al. Dissolved organic matter and inorganic ions in a central Himalayan glacier-insights into chemical composition and atmospheric sources. *Environ Sci Technol* 2013; **47**: 6181-88.
